# The genome sequence of the Heart Moth, *Dicycla oo *(Linnaeus 1758)

**DOI:** 10.12688/wellcomeopenres.19535.1

**Published:** 2023-07-21

**Authors:** Mark Sterling, David C. Lees, Natural History Museum Genome Acquisition Lab, Darwin Tree of Life Barcoding collective, Wellcome Sanger Institute Tree of Life programme, Wellcome Sanger Institute Scientific Operations: DNA Pipelines collective, Tree of Life Core Informatics collective, Darwin Tree of Life Consortium

**Affiliations:** 1 Natural History Museum, London, England, UK

**Keywords:** Dicycla oo, the Heart Moth, genome sequence, chromosomal, Lepidoptera

## Abstract

We present a genome assembly from an individual female *Dicycla oo* (the Heart Moth; Arthropoda; Insecta; Lepidoptera; Noctuidae). The genome sequence is 936.7 megabases in span. Most of the assembly is scaffolded into 31 chromosomal pseudomolecules, including the Z sex chromosome. The mitochondrial genome has also been assembled and is 15.29 kilobases in length. Gene annotation of this assembly on Ensembl identified 19,564 protein coding genes.

## Species taxonomy

Eukaryota; Metazoa; Ecdysozoa; Arthropoda; Hexapoda; Insecta; Pterygota; Neoptera; Endopterygota; Lepidoptera; Glossata; Ditrysia; Noctuoidea; Noctuidae; Xyleninae; Xylenini; Cosmiina; *Dicycla*; *Dicycla oo* (Linnaeus, 1758) (NCBI:txid1858094).

## Background

The Heart Moth, *Dicycla oo*, is a noctuid moth with a wingspan of 32–38 mm. The background colour is generally pale yellow (sometimes darker) with distinctive cross lines and dark lined pale yellow stigmata. It takes its English vernacular name from its distinctive and prominent heart shaped reniform stigma. The moth flies from mid-June to mid-July and is associated with woodland and parkland containing mature Pedunculate Oak ( *Quercus robur*). Unusually, the species comes to sugar lures well before dusk and although it is attracted to mercury vapour light, it usually does not arrive until the early hours of the morning; the larva feeds on oak ( Heath & Emmet, 1983), including *Q. pubescens* Willd. in Europe ( LepiForum, 2023). The handsome black, cream marked caterpillar (e.g. Lepiforum, 2023) is stated to be cannibalistic ( Wightman, 1931), as for members of the genus *Cosmia*. The moth oviposits in crevices in oak bark ( Lepiforum, 2023).

The Heart Moth has always been regarded as a rare species in the UK with a historical distribution in southern and central-eastern England ( NBN Atlas Partnership, 2021). It has declined greatly and Randle *et al.* (2019) state that it is now most frequent, albeit very locally, in Surrey and Berkshire. It may still occur in Northamptonshire. However, unpublished data provided to M. Sterling (February 2023) from the National Moth Recording Scheme, courtesy of Butterfly Conservation, contain only seven individual records from the UK since 2018, outside of one very restricted area of Surrey; these records being from West Sussex, Berkshire, and elsewhere in Surrey. In the UK the moth is classified as Red Data Book species (RDB3) and is a UK Biodiversity Action Plan species ( Parsons *et al.*, 2005) pursuant to Section 41 of the Natural Environment and Rural Communities Act (2006).

*Dicycla oo* occurs in the western Palearctic from Southern Scandinavia to the southern shores of the Mediterranean and with scattered records in western Asia ( GBIF Secretariat, 2023).

The genus *Dicycla* (Guenée, 1852) is monobasic. On BOLD (2023-03-02) the species formed a single DNA barcode cluster (BOLD:AAN1463) with an identical haplotype to public domain sequences from France and Italy, and its closest relatives among Noctuidae need to be clarified; *D. oo* exhibited at least 4.34% pairwise divergence from its nearest neighbour (BOLD:ACP3010) and 4.74% pairwise divergence from *Cosmia contusa* (Freyer, 1849).

The genome will be of utility in phylogenetic studies, including comparisons to genomic data for putative close relatives ( Boyes *et al*., 2022).

## Genome sequence report

**Figure 1.**
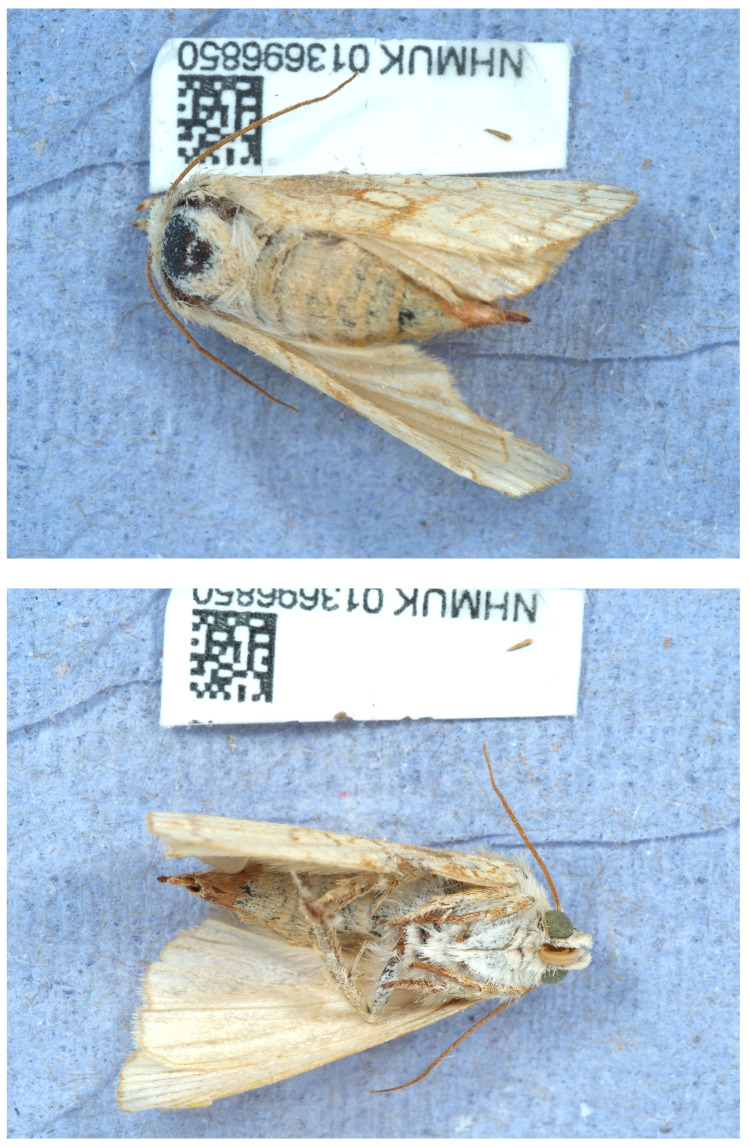
Photograph of the *Dicycla oo* (ilDicOoxx2) specimen used for genome sequencing.

The genome was sequenced from one female *Dicycla oo* ( Figure 1) collected in Surrey, UK. A total of 27-fold coverage in Pacific Biosciences single-molecule HiFi long reads was generated. Primary assembly contigs were scaffolded with chromosome conformation Hi-C data. Manual assembly curation corrected 37 missing joins or mis-joins and removed 11 haplotypic duplications, reducing the assembly length by 0.58% and the scaffold number by 11.83%, and increasing the scaffold N50 by 0.23%.

**Figure 2.**
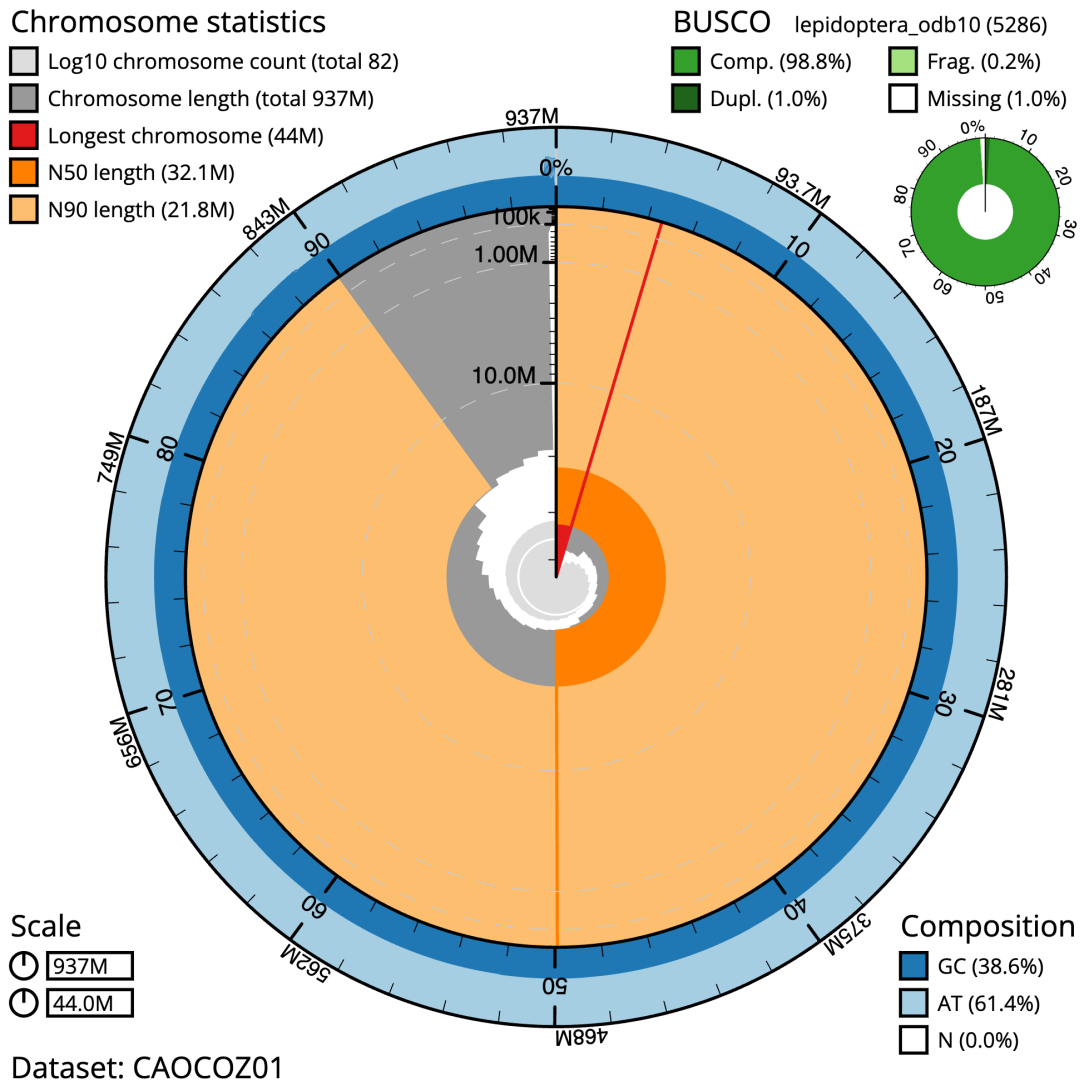
Genome assembly of *Dicycla oo*, ilDicOoxx2.1: metrics. The BlobToolKit Snailplot shows N50 metrics and BUSCO gene completeness. The main plot is divided into 1,000 size-ordered bins around the circumference with each bin representing 0.1% of the 936,717,301 bp assembly. The distribution of scaffold lengths is shown in dark grey with the plot radius scaled to the longest scaffold present in the assembly (43,981,956 bp, shown in red). Orange and pale-orange arcs show the N50 and N90 scaffold lengths (32,053,505 and 21,809,368 bp), respectively. The pale grey spiral shows the cumulative scaffold count on a log scale with white scale lines showing successive orders of magnitude. The blue and pale-blue area around the outside of the plot shows the distribution of GC, AT and N percentages in the same bins as the inner plot. A summary of complete, fragmented, duplicated and missing BUSCO genes in the lepidoptera_odb10 set is shown in the top right. An interactive version of this figure is available at https://blobtoolkit.genomehubs.org/view/ilDicOoxx2.1/dataset/CAOCOZ01/snail.

The final assembly has a total length of 936.7 Mb in 81 sequence scaffolds with a scaffold N50 of 32.1 Mb ( Table 1). Most (99.57%) of the assembly sequence was assigned to 31 chromosomal-level scaffolds, representing 30 autosomes and the Z sex chromosome. Chromosome-scale scaffolds confirmed by the Hi-C data are named in order of size ( Figure 2– Figure 5; Table 2). While not fully phased, the assembly deposited is of one haplotype. Contigs corresponding to the second haplotype have also been deposited. The mitochondrial genome was also assembled and can be found as a contig within the multifasta file of the genome submission.

**Table 1.** Genome data for *Dicycla oo*, ilDicOoxx2.1.

Project accession data		
Assembly identifier	ilDicOoxx2.1	
Species	*Dicycla oo*	
Specimen	ilDicOoxx2	
NCBI taxonomy ID	1858094	
BioProject	PRJEB58346	
BioSample ID	SAMEA111458042	
Isolate information	ilDicOoxx2, female: abdomen (DNA sequencing) ilDicOoxx1: leg (Hi-C scaffolding)	
Assembly metrics *		*Benchmark*
Consensus quality (QV)	64.9	*≥ 50*
*k*-mer completeness	100%	*≥ 95%*
BUSCO **	C:98.8%[S:97.9%,D:1.0%], F:0.2%,M:1.0%,n:5,286	*C ≥ 95%*
Percentage of assembly mapped to chromosomes	99.57%	*≥ 95%*
Sex chromosomes	Z chromosome	*localised homologous pairs*
Organelles	Mitochondrial genome assembled	*complete single alleles*
Raw data accessions		
PacificBiosciences SEQUEL II	ERR10677857	
Hi-C Illumina	ERR10684085	
Genome assembly		
Assembly accession	GCA_948252095.1	
*Accession of alternate haplotype*	GCA_948295755.1	
Span (Mb)	936.7	
Number of contigs	201	
Contig N50 length (Mb)	12.6	
Number of scaffolds	81	
Scaffold N50 length (Mb)	32.1	
Longest scaffold (Mb)	44.0	
Genome annotation		
Number of protein-coding genes	19,564	
Number of gene transcripts	19,734	

* Assembly metric benchmarks are adapted from column VGP-2020 of “Table 1: Proposed standards and metrics for defining genome assembly quality” from ( Rhie *et al.*, 2021). ** BUSCO scores based on the lepidoptera_odb10 BUSCO set using v5.3.2. C = complete [S = single copy, D = duplicated], F = fragmented, M = missing, n = number of orthologues in comparison. A full set of BUSCO scores is available at https://blobtoolkit.genomehubs.org/view/ilDicOoxx2.1/dataset/CAOCOZ01/busco.

**Figure 3.**
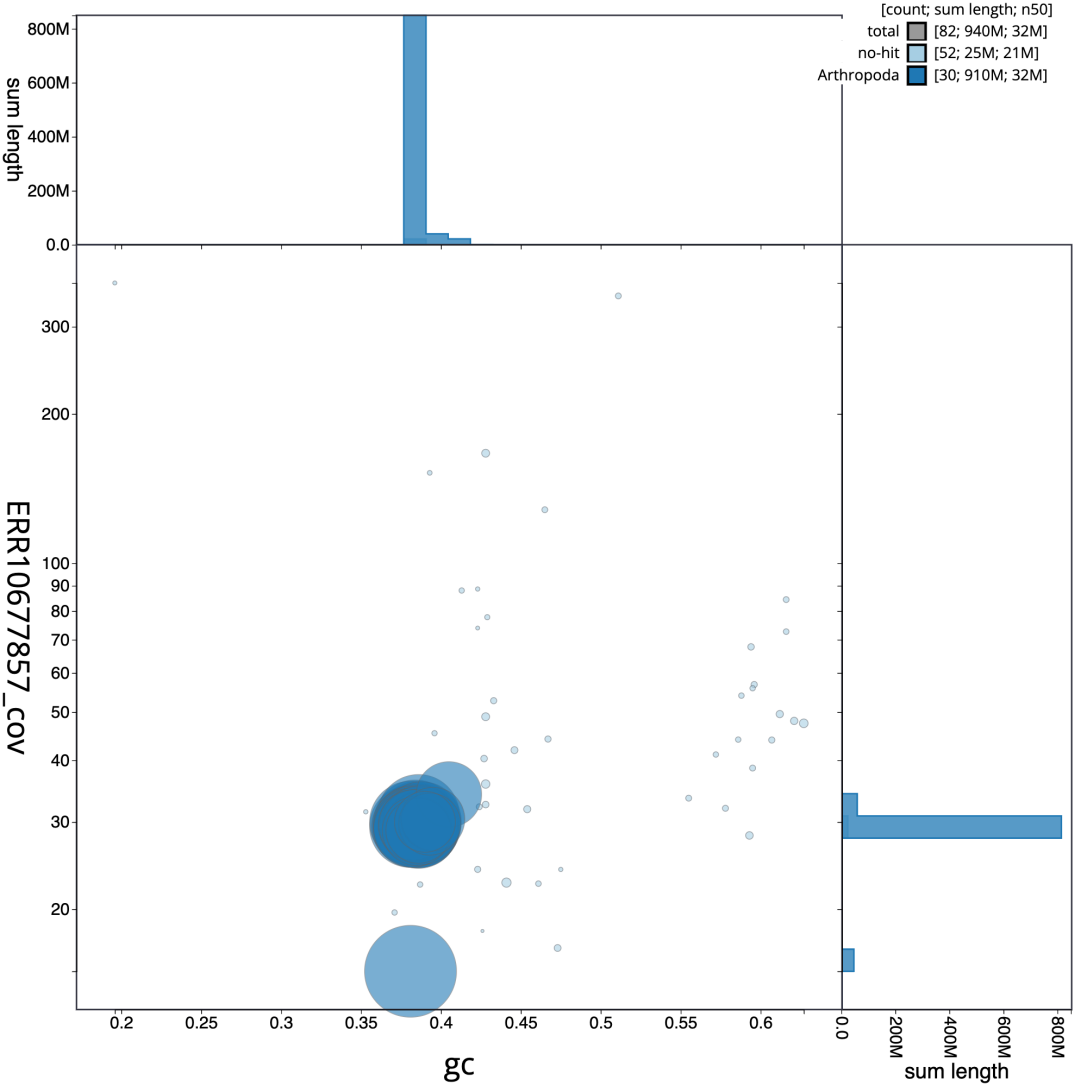
Genome assembly of *Dicycla oo*, ilDicOoxx2.1: BlobToolKit GC-coverage plot. Scaffolds are coloured by phylum. Circles are sized in proportion to scaffold length. Histograms show the distribution of scaffold length sum along each axis. An interactive version of this figure is available at https://blobtoolkit.genomehubs.org/view/ilDicOoxx2.1/dataset/CAOCOZ01/blob.

**Figure 4.**
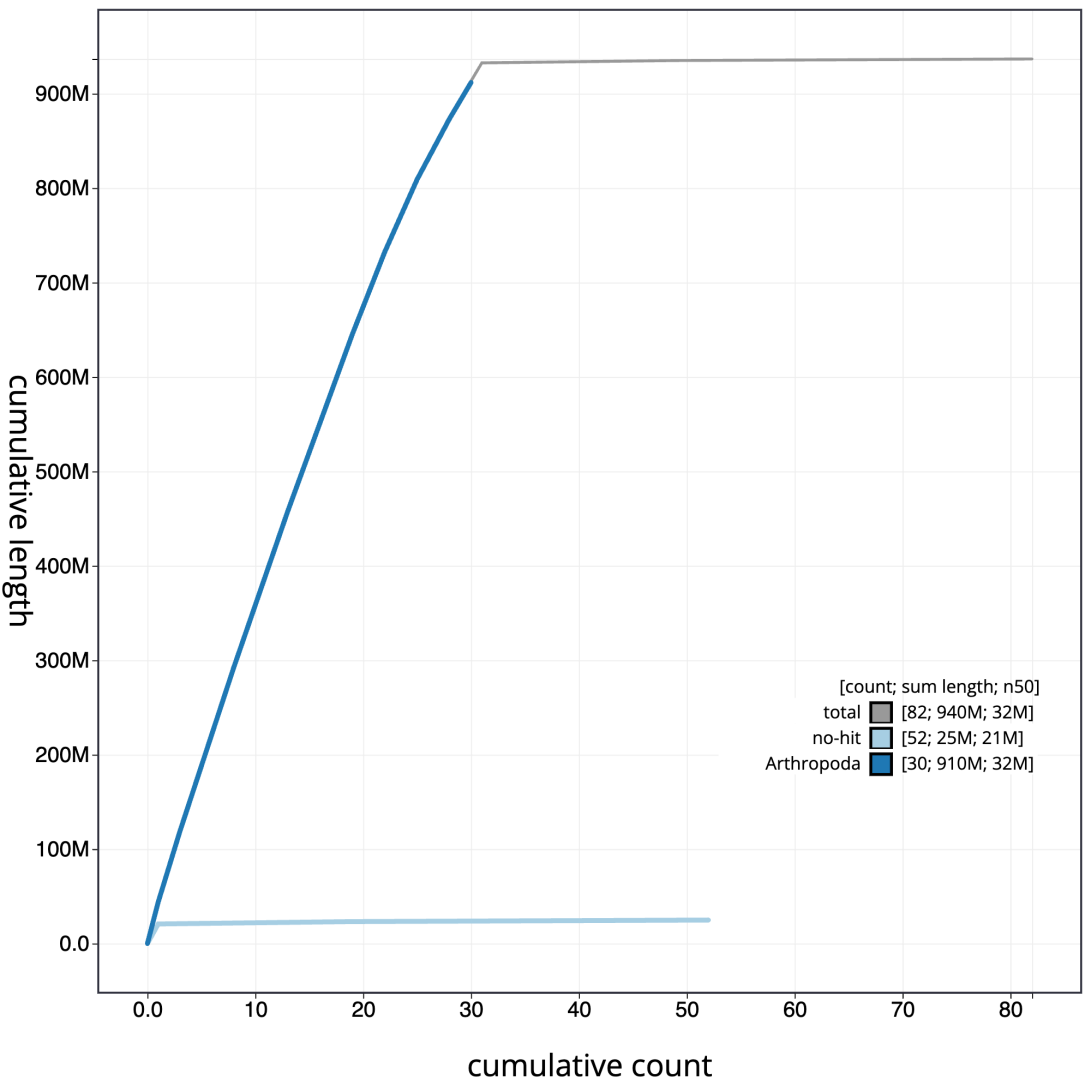
Genome assembly of *Dicycla oo*, ilDicOoxx2.1: BlobToolKit cumulative sequence plot. The grey line shows cumulative length for all scaffolds. Coloured lines show cumulative lengths of scaffolds assigned to each phylum using the buscogenes taxrule. An interactive version of this figure is available at https://blobtoolkit.genomehubs.org/view/ilDicOoxx2.1/dataset/CAOCOZ01/cumulative.

**Figure 5.**
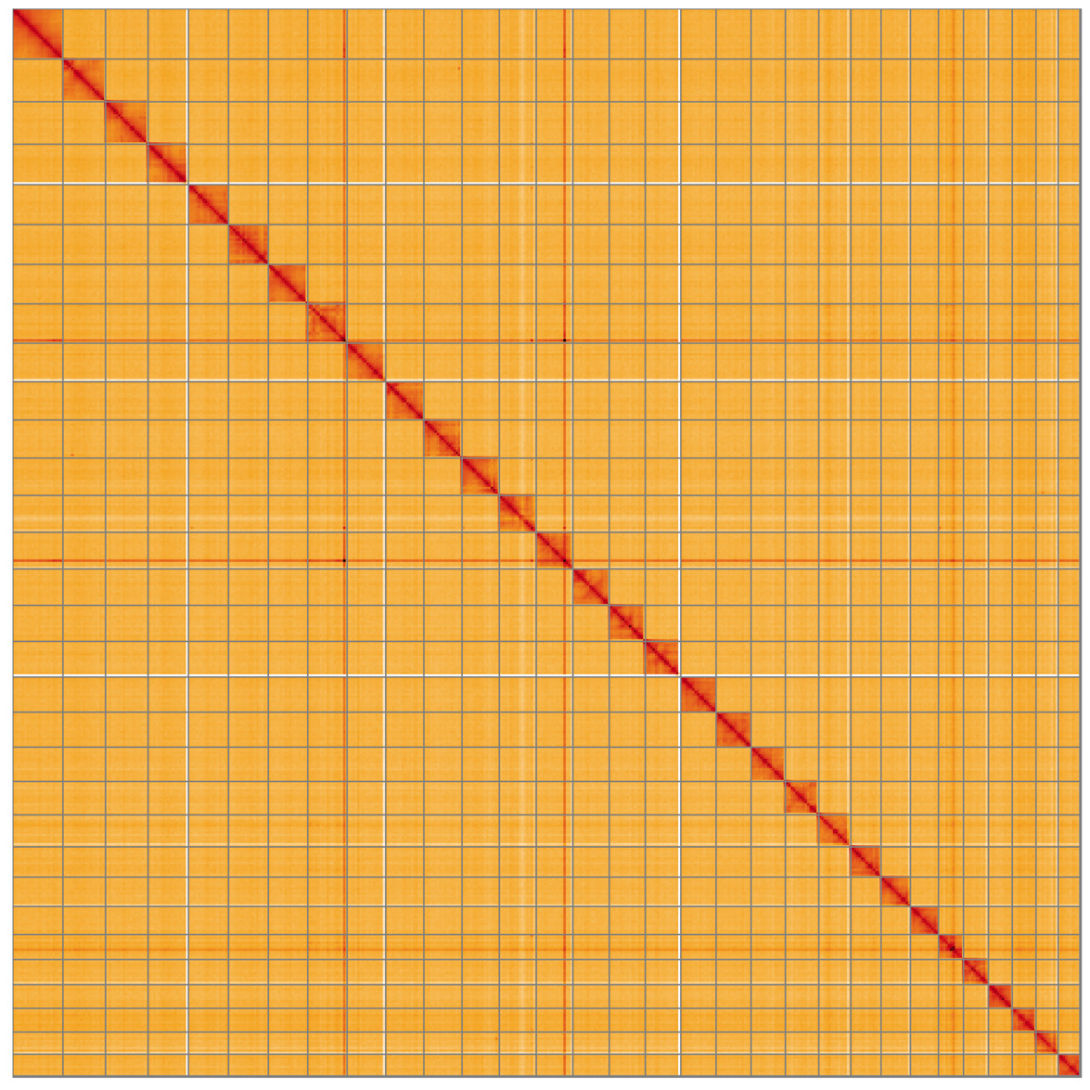
Genome assembly of *Dicycla oo*, ilDicOoxx2.1: Hi-C contact map of the ilDicOoxx2.1 assembly, visualised using HiGlass. Chromosomes are shown in order of size from left to right and top to bottom. An interactive version of this figure may be viewed at https://genome-note-higlass.tol.sanger.ac.uk/l/?d=HbvMXnQDS5SNvmlDeT_N6Q.

**Table 2.** Chromosomal pseudomolecules in the genome assembly of *Dicycla oo*, ilDicOoxx2.

INSDC accession	Name	Length (Mb)	GC%
OX411710.1	1	37.34	38.5
OX411711.1	2	37.11	38.5
OX411712.1	3	35.26	38.5
OX411713.1	4	35	38.5
OX411714.1	5	34.65	38
OX411715.1	6	34.56	38
OX411716.1	7	34.36	38.5
OX411717.1	8	33.7	38.5
OX411718.1	9	33.43	38.5
OX411719.1	10	33.41	38.5
OX411720.1	11	32.51	38.5
OX411721.1	12	32.23	38.5
OX411722.1	13	32.05	39
OX411723.1	14	31.87	38
OX411724.1	15	31.38	38
OX411725.1	16	31.02	38
OX411726.1	17	30.82	38
OX411727.1	18	30.64	38
OX411728.1	19	29.95	38.5
OX411729.1	20	29.21	38.5
OX411730.1	21	27.89	39
OX411731.1	22	26.43	38.5
OX411732.1	23	25.76	38.5
OX411733.1	24	24.59	38.5
OX411734.1	25	21.82	40.5
OX411735.1	26	21.81	38.5
OX411736.1	27	20.76	38.5
OX411737.1	28	20.62	39.5
OX411738.1	29	19.59	39.5
OX411739.1	30	18.81	39
OX411709.1	Z	43.98	38
OX411740.1	MT	0.02	20

The estimated Quality Value (QV) of the final assembly is 64.9 with *k*-mer completeness of 100%, and the assembly has a BUSCO v5.3.2 completeness of 98.8% (single = 97.9%, duplicated = 1.0%), using the lepidoptera_odb10 reference set ( *n* = 5,286).

Metadata for specimens, spectral estimates, sequencing runs, contaminants and pre-curation assembly statistics can be found at https://links.tol.sanger.ac.uk/species/1858094.

## Genome annotation report

The *Dicycla oo* genome assembly (GCA_948252095.1) was annotated using the Ensembl rapid annotation pipeline ( Table 1; https://rapid.ensembl.org/Dicycla_oo_GCA_948252095.1/Info/Index). The resulting annotation includes 19,734 transcribed mRNAs from 19,564 protein-coding genes.

## Methods

### Sample acquisition and nucleic acid extraction

The *Dicycla oo* specimens used for genome sequencing (specimen ID NHMUK013696850, individual ilDicOoxx2) and Hi-C scaffolding (NHMUK013696844, individual ilDicOoxx1) were collected from Surrey (further details of locality redacted) on 2021-07-16. The specimens were collected and identified by Mark Sterling and David Lees (Natural History Museum). The specimen was retained for eggs and subsequently preserved by dry freezing at –80°C on 2021-07-23.

The ilDicOoxx2 specimen was prepared for DNA extraction at the Tree of Life laboratory, Wellcome Sanger Institute (WSI). The sample was weighed and dissected on dry ice. The abdomen tissue was disrupted using a Nippi Powermasher fitted with a BioMasher pestle. DNA was extracted at the WSI Scientific Operations core using the Qiagen MagAttract HMW DNA kit, according to the manufacturer’s instructions.

### Sequencing

Pacific Biosciences HiFi circular consensus DNA sequencing libraries were constructed according to the manufacturers’ instructions. DNA sequencing was performed by the Scientific Operations core at the WSI on the Pacific Biosciences SEQUEL II (HiFi) instrument. Hi-C data were also generated from the leg tissue of ilDicOoxx1 using the Arima2 kit and sequenced on the Illumina NovaSeq 6000 instrument.

### Genome assembly, curation and evaluation

Assembly was carried out with Hifiasm ( Cheng *et al.*, 2021) and haplotypic duplication was identified and removed with purge_dups ( Guan *et al.*, 2020). The assembly was then scaffolded with Hi-C data ( Rao *et al.*, 2014) using YaHS ( Zhou *et al.*, 2023). The assembly was checked for contamination and corrected as described previously ( Howe *et al.*, 2021). Manual curation was performed using HiGlass ( Kerpedjiev *et al.*, 2018) and Pretext ( Harry, 2022). The mitochondrial genome was assembled using MitoHiFi ( Uliano-Silva *et al.*, 2022), which runs MitoFinder ( Allio *et al.*, 2020) or MITOS ( Bernt *et al.*, 2013) and uses these annotations to select the final mitochondrial contig and to ensure the general quality of the sequence.

A Hi-C map for the final assembly was produced using bwa-mem2 ( Vasimuddin *et al.*, 2019) in the Cooler file format ( Abdennur & Mirny, 2020). To assess the assembly metrics, the *k*-mer completeness and QV consensus quality values were calculated in Merqury ( Rhie *et al.*, 2020). This work was done using Nextflow ( Di Tommaso *et al.*, 2017) DSL2 pipelines “sanger-tol/readmapping” ( Surana *et al.*, 2023a) and “sanger-tol/genomenote” ( Surana *et al.*, 2023b). The genome was analysed within the BlobToolKit environment ( Challis *et al.*, 2020) and BUSCO scores ( Manni *et al.*, 2021; Simão *et al.*, 2015) were calculated.

Table 3 contains a list of relevant software tool versions and sources.

**Table 3.** Software tools: versions and sources.

Software tool	Version	Source
BlobToolKit	4.1.5	https://github.com/blobtoolkit/blobtoolkit
BUSCO	5.3.2	https://gitlab.com/ezlab/busco
Hifiasm	0.16.1-r375	https://github.com/chhylp123/hifiasm
HiGlass	1.11.6	https://github.com/higlass/higlass
Merqury	MerquryFK	https://github.com/thegenemyers/MERQURY.FK
MitoHiFi	2	https://github.com/marcelauliano/MitoHiFi
PretextView	0.2	https://github.com/wtsi-hpag/PretextView
purge_dups	1.2.3	https://github.com/dfguan/purge_dups
sanger-tol/genomenote	v1.0	https://github.com/sanger-tol/genomenote
sanger-tol/readmapping	1.1.0	https://github.com/sanger-tol/readmapping/tree/1.1.0
YaHS	1.2a	https://github.com/c-zhou/yahs

### Genome annotation

The BRAKER2 pipeline ( Brůna *et al.*, 2021) was used in the default protein mode to generate annotation for the *Dicycla oo* assembly (GCA_948252095.1) in Ensembl Rapid Release.

### Wellcome Sanger Institute – Legal and Governance

The materials that have contributed to this genome note have been supplied by a Darwin Tree of Life Partner. The submission of materials by a Darwin Tree of Life Partner is subject to the **‘Darwin Tree of Life Project Sampling Code of Practice’**, which can be found in full on the Darwin Tree of Life website here. By agreeing with and signing up to the Sampling Code of Practice, the Darwin Tree of Life Partner agrees they will meet the legal and ethical requirements and standards set out within this document in respect of all samples acquired for, and supplied to, the Darwin Tree of Life Project.

Further, the Wellcome Sanger Institute employs a process whereby due diligence is carried out proportionate to the nature of the materials themselves, and the circumstances under which they have been/are to be collected and provided for use. The purpose of this is to address and mitigate any potential legal and/or ethical implications of receipt and use of the materials as part of the research project, and to ensure that in doing so we align with best practice wherever possible. The overarching areas of consideration are:

- Ethical review of provenance and sourcing of the material
- Legality of collection, transfer and use (national and international)

Each transfer of samples is further undertaken according to a Research Collaboration Agreement or Material Transfer Agreement entered into by the Darwin Tree of Life Partner, Genome Research Limited (operating as the Wellcome Sanger Institute), and in some circumstances other Darwin Tree of Life collaborators.

## Data Availability

European Nucleotide Archive: *Dicycla oo*. Accession number PRJEB58346; https://identifiers.org/ena.embl/PRJEB58346. ( Wellcome Sanger Institute, 2022) The genome sequence is released openly for reuse. The *Dicycla oo* genome sequencing initiative is part of the Darwin Tree of Life (DToL) project. All raw sequence data and the assembly have been deposited in INSDC databases. Raw data and assembly accession identifiers are reported in Table 1.

## Author information

Members of the Natural History Museum Genome Acquisition Lab are listed here: https://doi.org/10.5281/zenodo.4790042.

Members of the Darwin Tree of Life Barcoding collective are listed here: https://doi.org/10.5281/zenodo.4893703.

Members of the Wellcome Sanger Institute Tree of Life programme are listed here: https://doi.org/10.5281/zenodo.4783585.

Members of Wellcome Sanger Institute Scientific Operations: DNA Pipelines collective are listed here: https://doi.org/10.5281/zenodo.4790455.

Members of the Tree of Life Core Informatics collective are listed here: https://doi.org/10.5281/zenodo.5013541.

Members of the Darwin Tree of Life Consortium are listed here: https://doi.org/10.5281/zenodo.4783558.
