# Supplementary material for: The genome sequence of the Heart Moth, Dicycla oo (Linnaeus 1758)
Source: Wellcome Open Res. 2023 Jul 21;8:318. [Version 1] doi: 10.12688/wellcomeopenres.19535.1 (PMC11292181; doi:10.12688/wellcomeopenres.19535.1)
Supplement: Peer review history

# Reviewer report: Reviewer response for version 1

**DOI:** 10.21956/wellcomeopenres.21639.r89210

**Published:** 2024-07-31

**Authors:** Jerome H L Hui

**Affiliations:** 1 The Chinese University of Hong Kong, Hong Kong, Hong Kong

In this data note, Sterling, Lees, and colleagues report the genome sequence of a female heart moth *Dicycla oo *(Linnaeus, 1758). According to the GBIF and UKMoths, this is a species that can be found in many places in South England. Prior to this report, available molecular data of this species are mainly mitochondrial cytochrome oxidase subunit 1 (COI) gene sequences deposited to the NCBI database. This new genome resource is useful for further studies, such as understanding the impact of climate change on this species, as well as ecological, evolutionary, and genomics questions related to insects more widely.

 This genome resource is excellent from the summary statistics, with high BUSCO number scores, high sequence continuity (scaffold N50), and majority of sequences contained on the 30 pseudochromosomes (plus sex chromosome and mitochondrion). All in all, this is a valuable contribution.

Are sufficient details of methods and materials provided to allow replication by others?

Yes

Is the rationale for creating the dataset(s) clearly described?

Yes

Are the datasets clearly presented in a useable and accessible format?

Yes

Are the protocols appropriate and is the work technically sound?

Yes

Reviewer Expertise:

Genomics, evolution, invertebrates

I confirm that I have read this submission and believe that I have an appropriate level of expertise to confirm that it is of an acceptable scientific standard.

# Reviewer report: Reviewer response for version 1

**DOI:** 10.21956/wellcomeopenres.21639.r88147

**Published:** 2024-07-09

**Authors:** Chiara Bortoluzzi

**Affiliations:** 1 University of Florence, Florence, Italy

In this genome note, the Darwin Tree of Life consortium report the sequencing of the Heart Moth  ( *Dicycla oo*) genome, a species of moth endemic to the United Kingdom. This genome is a high quality one and will play a significant role in future phylogenetic studies.

 The report is very well written and all datasets are presented in a clear manner. The data is readily accessible to the public through both ENA, NCBI, and Ensembl Rapid Release, with all provided links functioning properly. The values presented in tables are accurate and align with the information available in public repositories. I did not find any major issues  and I would thus like to recommend this Genome Note for indexing in its current form.

Are sufficient details of methods and materials provided to allow replication by others?

Yes

Is the rationale for creating the dataset(s) clearly described?

Yes

Are the datasets clearly presented in a useable and accessible format?

Yes

Are the protocols appropriate and is the work technically sound?

Yes

Reviewer Expertise:

Biodiversity conservation, comparative genomics, population genomics

I confirm that I have read this submission and believe that I have an appropriate level of expertise to confirm that it is of an acceptable scientific standard.
